# Artificial intelligence based optimization with deep learning model for blockchain enabled intrusion detection in CPS environment

**DOI:** 10.1038/s41598-022-17043-z

**Published:** 2022-07-28

**Authors:** Romany F. Mansour

**Affiliations:** Department of Mathematics, Faculty of Science, New Valley University, El-Kharga, 72511 Egypt

**Keywords:** Engineering, Mathematics and computing

## Abstract

Cyber physical system (CPS) is a network of cyber and physical elements, which interact with one another in a feedback form. CPS approves critical infrastructure and is treated as essential in day to day since it forms the basis of futuristic smart devices. An increased usage of CPSs poses security as a challenging issue and intrusion detection systems (IDS) can be applied for the identification of network intrusions. The latest advancements in the field of artificial intelligence (AI) and deep learning (DL) enables to design effective IDS models for the CPS environment. At the same time, metaheuristic algorithms can be employed as a feature selection approach in order to reduce the curse of dimensionality. With this motivation, this study develops a novel Poor and Rich Optimization with Deep Learning Model for Blockchain Enabled Intrusion Detection in CPS Environment, called PRO-DLBIDCPS technique. The proposed PRO-DLBIDCPS technique initially introduces an Adaptive Harmony Search Algorithm (AHSA) based feature selection technique for proper selection of feature subsets. For intrusion detection and classification, and attention based bi-directional gated recurrent neural network (ABi-GRNN) model is applied. In addition, the detection efficiency of the ABi-GRNN technique has been enhanced by the use of Poor and rich optimization (PRO) algorithm based hyperparameter optimizer, which resulted in enhanced intrusion detection results. Furthermore, blockchain technology is applied for enhancing security in the CPS environment. In order to demonstrate the enhanced outcomes of the PRO-DLBIDCPS technique, a wide range of simulations was carried out on benchmark dataset and the results reported the better outcomes of the PRO-DLBIDCPS technique in terms of several measures.

## Introduction

**Figure 1:**
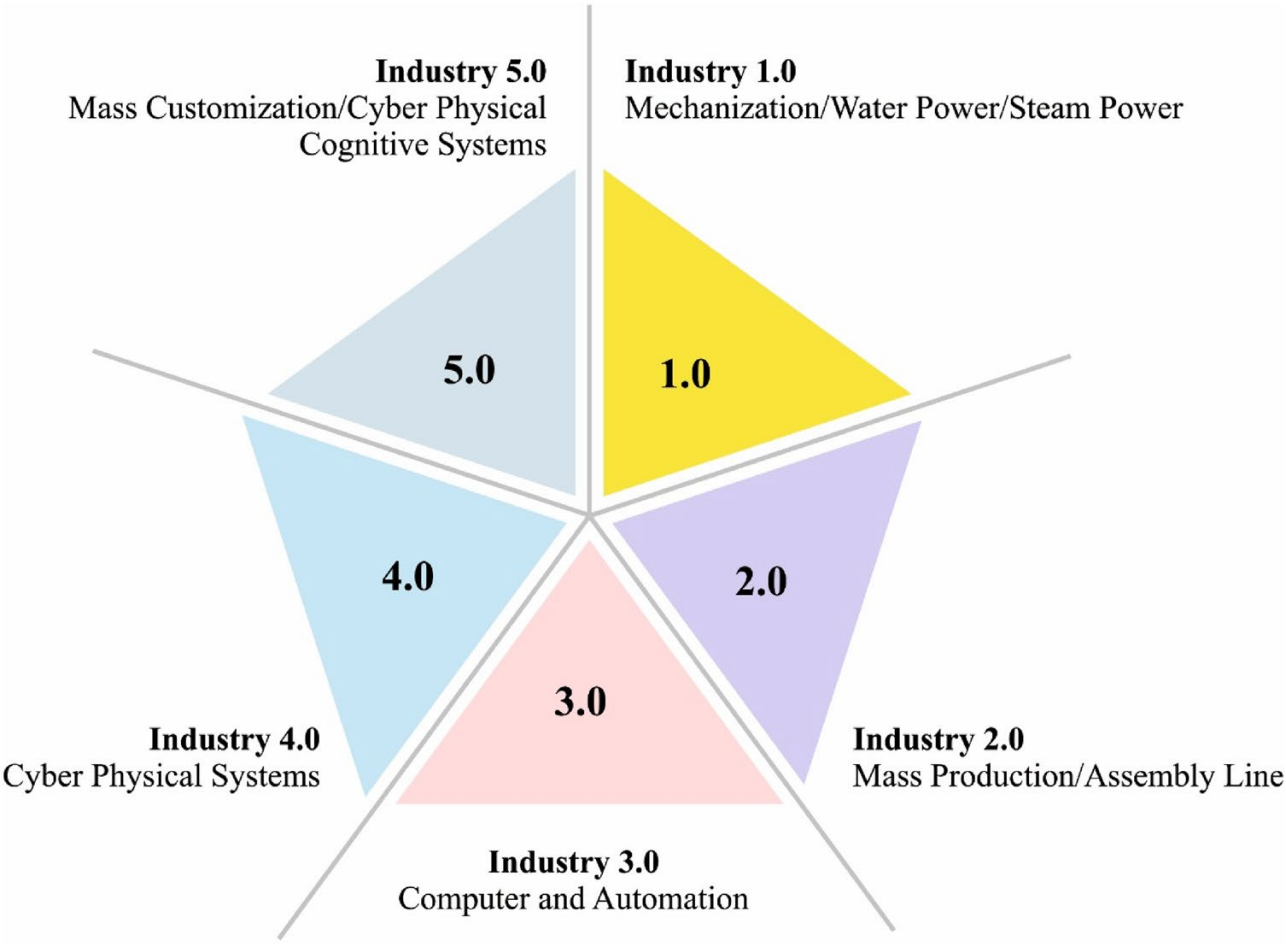
Evolution of CPS in Industry 4.0.

Cyber physical system (CPS) is a multi-dimensional and complex scheme, which incorporates industrial component over the Internet of Things (IoT) to construct effective CPS production environments^1^. In addition, CPS can be utilized in various applications such as health care, smart transportation, and smart homes. The evolution of CPS in the industry 4.0 is depicted in Fig. 1. A CPS includes an integration of physical and logical systems for including transmission among human, analog, and digital components. CPS network is contained of networking modules, sensors, and actuators, that is appropriate in the automation, power, civil structure, medicine, and development field, amongst other^2^. In general, a CPS is a complex scheme where cyber applications and external operations are supported in an integrated manner. Even though information and communication technology (ICT) is very progressive in CPS, but still cybersecurity is taken into account as a major problem in various fields. The most complex vulnerability in CPS is intrusion hazard. Over the previous years, special consideration has been given to the improvement of CPS security.

Intrusion detection (ID) is a significant application to maximize the integrity of CPS. Usually, an Intrusion detection system (IDS) is employed for efficiently preventing attack^3^. IDS approach is generally classified into two main categories: anomaly and misuse predictions. Firstly, the feature of familiar attacks is employed for misuse prediction. Currently, the audited information is associated with a dataset and described as an intrusion. Even though misuse detector generates the minimal FP, this detector has enormous limitation^4^. Intrusion Detection System (IDS), identify intrusion that other security approaches are unable to avoid, worked as another line of defence, and have a considerable role in safeguarding CPS^5,6^. IDS based anomaly detection construct a usual behavior profile and categorize behavior that don’t matching this usual profile as attack^7^. Compared with other methods, anomaly-detection IDS could identify unknown attack that is a crucial feature for CPS. It connects a wide-ranging device with distinct computational resources, battery capacity, transmission technology, operating, and software system. This heterogeneity challenging the placement of security solution and increase the attack surface, which makes CPS very susceptible to unknown and new attacks^8^.

Conventional machine learning (ML) approaches have been demonstrated for identifying data patterns and detecting cyberattacks effectively in IDS. But they revealed to not scale efficiently with huge data sets and to accomplish lower performance for detecting cyberattack once network node is distributed considerably^9^. At the same time, advancement in deep learning (DL) stimulates IDS mechanism able to handle the current cyberattack, level of complexity and sophistication^10^. Meng et al.^11^ focuses on the finding of the malicious device and designed a trust-related IDS based behavioral profiling. A node reputation is considered by recognizing the variance in Euclidean distance between two behavioural profiles. Xing et al.^12^ proposed an effective trust-based IDS for autonomous vehicular networks (AVN). Firstly, proposed a trust assessment method for autonomous driving vehicles (ADV), where each trust assessment data of a provided ADV is exploited for computing its trust value. Next, depending on the trust assessment method, a two-phase IDS has been introduced. Next, presented a reinforcement-learning-based incentive model for stimulating ADV to report a warning.

Arshad et al.^13^ projected an IDS for the energy-constraint IoT device that forms the basis of the industrial internet of things (IIoT) ecosystem. Considering the adhoc nature of this scheme and emergent difficult threats includes botnets, we estimate the opportunity of collaborations among the edge devices and the host (IoT device) for successful IDS while minimalizing transmission overhead and power utilization. Wang et al.^14^ designed a combined deep IDS for overcoming the low classification accuracy and longer trained time of the current deep neural network (DNN) system and to accomplish appropriate response to intrusion behavior. For host IDS, a combined deep IDS based deep belief network (DBN) Softmax is created. Simultaneously, a smaller batch gradient descent approach is utilized for network optimization and training. Ibor et al.^15^ presented a hybrid model for intrusion prediction on CPS transmission network. Next, utilize a bio-inspired hyperparameter searching method for generating an enhanced DNN model based on the core hyperparameter of a neural network. Moreover, derived a predictive system based enhanced neural network (NN) system.

This study develops a novel poor and rich optimization with a deep learning model for blockchain enabled intrusion detection in a CPS environment, called the PRO-DLBIDCPS technique. The proposed PRO-DLBIDCPS technique designs an adaptive harmony search algorithm based feature selection (AHSA) technique. Besides, Poor and rich optimization (PRO) algorithm with an attention based bi-directional gated recurrent neural network (ABi-GRNN) model are utilized to detect and classify intrusions. Moreover, blockchain technology is applied for enhancing security in the CPS environment. A wide ranging simulation analysis is performed to ensure the enhanced performance of the PRO-DLBIDCPS technique in terms of several measures.

## Methods

**Figure 2:**
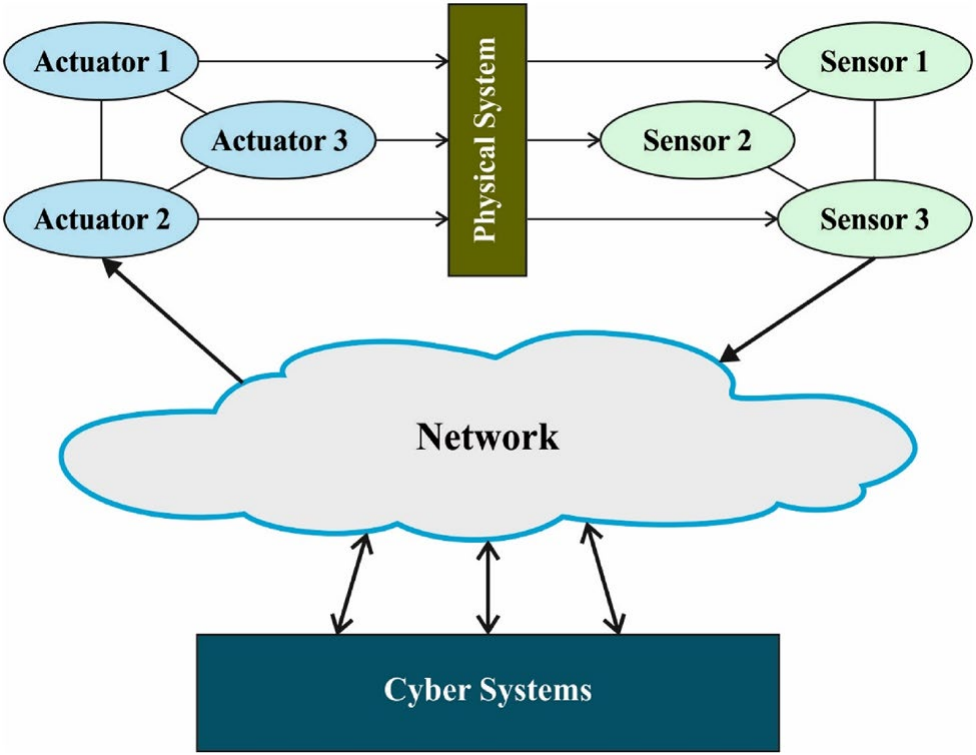
System architecture of CPS.

In this study, a new PRO-DLBIDCPS technique has been developed for intrusion detection in the CPS environment. The PRO-DLBIDCPS technique encompasses different processes namely pre-processing, AHSA for election of features, ABi-GRNN classifier, and PRO hyperparameter optimizer. The detection efficiency of the ABi-GRNN technique has been enhanced by the use of PRO algorithm based hyperparameter optimizer, which results in enhanced intrusion detection results. The overall system architecture is shown in Fig. 2.

### Blockchain technology

**Figure 3:**
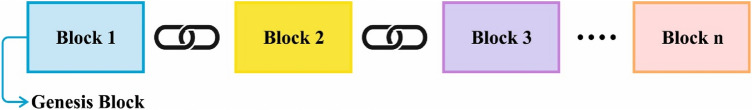
Blockchain architecture.

In this work, blockchain technology is included to boost security in the CPS environment. A blockchain is an immutable distributed dataset where new time-stamped transaction is grouped and appended into a hash-chain of block^16^. The structure of the blockchain is given in Fig. 3. The fundamental blockchain protocols define how many copies of the block could be maintained and constructed in a distributed manner. A crucial factor of this protocol is deciding how a network of participants, called a miner, could determine consensus on the present state of the blockchain. There are distinct kinds of blockchain architecture (that is., private, public, permissioned, and permission-less). There are two prevalent approaches for the same, such as Proof of Stake (PoS) and Proof of Work (PoW). When this task is completed, the new transaction is added to the blockchain. All the blocks contain a unique code named a hash, which contain hash of the preceding block in the chain, and is utilized for connecting the block together in a certain order. Some miner should implement a set of computations for establishing the credibility as a leader. This computation resolves a puzzle for mapping random sized data to a fixed size. In other networks, a leader could be selected in one of these two methods. In Proof of Work (PoW), several miner tries to resolve the puzzle and the one that finished first, broadcasted to the group proof that the work is completed. Then, other miner validates that the work completed is correct. When each one verifies this, they choose that certain miner as the leader. The main goal of the block is to preserve a list of confirmed transaction with a cryptographic hash function. The hash function is effective due to the subsequent property.

- It produces an output of fixed length regardless of the input length.
- It is deterministic that generate similar output for a provided input.
- It is irrevocable that getting similar input from the output is impossible.
- Any perturbation to the original input generates new output.
- The hash computation is faster with minimal overhead.

The block in the blockchain is connected to the initial genes is block and confirmed by the hashes. Each block is linked via the relationship of each hash, which implies all the blocks have the prior hash, and further get hashed in the following block. Any such modification to the hash causes the chain to be broken since the original hash is attached to the following block in the chain. Recalculate the original hash for restoring the chain require a massive number of computational power. Additionally, the nonce is added thus the miner plays with the data to generate a hash that output three zeroes. When the miner has found a nonce that leads to block hash being under the difficult threshold, finally considered the block is valid, and it is broadcasted to the network.

As blockchain can protect the integrity of data storage and ensure process transparency, it has a potential to be applied to intrusion detection domain^17^. The lack of universal trust implies a need for a distributed consensus mechanism for block validation in blockchain networks. Blockchain based Anamoly detection approaches have been used to enhance security. For details refer^18,19^.

### Design of AHSA-FS technique

Primarily, the networking data is pre-processed and is passed into the AHSA-FS technique for choosing feature subsets. HSA is a metaheuristic algorithm, stimulated from the basic principle of the musician’s inventiveness of the harmony. The control parameters were bandwidth (BW), harmony memory consideration rate (HMCR), and pitch adjustment rate (PAR). In this method, the length of harmony was the amount of samples chosen in the data set. It utilizes a real encoded model for representing all bits of harmony^20^. To harmony vector representation, all bits are allocated with the real number drawn in the search with lower bound and upper bound with the total number of features (TNF) and rounded for integrating value demonstrating the feature index. Supposing when harmony length is 10 afterward all bits are signified with arbitrary real number amongst l to TNF. The fitness value of all harmony is estimated consider classification error as FF signified as:

1 $$ Fitness{\text{-}}Vector = fit\left( {Value_{1} } \right),fit\left( {Value_{2} } \right),L,fit\left( {V_{HMS} } \right) $$

where, $fit\left({Value}_{1}\right)$ and $fit\left({Value}_{2}\right) \text{are fitness values},\text{ and } HMS\text{ is harmony memory size}$. The current harmony was improvised from the subsequent ways for *j* $=1$ to *N,* where* N* refers to the size of population*.*

if $$(rand(O, 1)< HMCR)$$

2 $${g}_{new}\left(j\right)={g}_{f}$$

where $g$ signifies the all sample index and $f=\text{1,2},3,\cdots ,HMS$

if $$(rand(O, 1)<PAR)$$

3 $${g}_{new}\left(\text{j}\right)={g}_{new}\left(\text{j}\right)+BW$$

4 $$else\;{g}_{new}(\text{j})=LB(\text{j})+rand(O, 1)\times (UB(\text{j})-LB\left(\text{j}\right)$$

At this point, the float number optimized technique was utilized to feature representations. Thus, all index from the harmony represents a feature. According to this probability of features from the feature subset, feature index was computed utilizing distribution factors under all samples ration improvisation signified as $F{D}_{j}^{g}$ is provided by

5 $$F{D}_{j}^{g}=a*\left(\frac{P{D}_{j}}{P{D}_{j}+N{D}_{j}}\right)+\left(1-\frac{\left(P{D}_{j}+N{D}_{j}\right)}{\text{max}\left(P{D}_{j}+N{D}_{j}\right)}\right)$$

where $P{D}_{j}$ refers the amount of times feature $j$ is approaching in optimum and $N{D}_{j}$ defined the amount of times feature $j$ was coming in a worse subset. The superior probability feature is maximum possibilities to come from the last subset. Eventually, according to the end condition (the amount of iterations or tolerable classification error) improvisation was completed and decreased the group of features is chosen under this phase. When $fit({g}_{new}(\text{j})$ is superior to fir $(worst)$ afterward upgrade harmony as:

6 $${g}_{worst}={g}_{new}$$

The last of all iterations the parameters PAR, HMCR, and BW are altered from the subsequent manner

7 $$HMC{R}_{t}=\frac{1}{1+{e}^{-HMC{R}_{t}}}, PA{R}_{t}=\frac{1}{1+{e}^{-PA{R}_{t}}}, BV{V}_{f}=\frac{1}{1+{e}^{-B{W}_{t}}}$$

Utilizing in Eq. (7), a sigmoidal transformation was executed to these components for bringing the value as to range. This work illustrates the multistage FS technique executing the benefits of both wrapper and filter approaches.

### Design of ABi-GRNN technique

At the time of intrusion detection and classification process, the ABi-GRNN technique has been developed for the identification of intrusions in the CPS environment. The conventional recurrent neural network (RNN) model handles the sequence problem with the utilization of earlier data based on the forward input series and does not considers the succeeding data. For resolving this issue, the BiRNN model has been developed by memorizing the previous and latter data. The major concept is to utilize a pair of RNNs in processing forward as well as reverse sequences, respectively. The outcome is afterward linked to the identical output layer and thereby the bi-directional contextual data for the feature sequence can be saved^21^. The Bi-GRNN technique is attained by replacing the hidden layer neuron in the BiRNN with GRU memory unit. At time $t$, the hidden layer of Bi-GRNN provides ${h}_{t}$ and it can be computed using Eqs. (8)–(10):

8 $$\overrightarrow{{h}_{t}}=\sigma \left({W}_{x\overrightarrow{h}}{x}_{t}+{W}_{\overrightarrow{h}\overrightarrow{h}}\overrightarrow{{h}_{t-1}}+{b}_{\overrightarrow{h}}\right)$$

9 $$\overleftarrow{{h}_{t}}=\sigma ({W}_{x\overleftarrow{h}}{x}_{t}+{W}_{\overleftarrow{h}\overleftarrow{h}}\overleftarrow{{h}_{t-1}}+{b}_{\overleftarrow{h}})$$

10 $${h}_{t}=\overrightarrow{{h}_{t}}\oplus \overleftarrow{{h}_{t}}$$

where $W$ implies weight matrix linking a pair of layers, $b$ denotes the bias vector, $\sigma$ indicates activation function, $\overrightarrow{{h}_{t}}$ and $\overleftarrow{{h}_{t}}$ indicates the outcome of positive and negative GRU respectively. $\oplus is$ element‐wise sum.

The attention method was utilized in the ABi-GRNN technique for representing the correlation between the data and output. This technique was primary implemented for the task of machine translations. The feed‐forward attention technique adapted during this case is a direct simplification of the convention attention system. The simplification technique is for constructing a single vector $\mathcal{C}$ in the complete order, generated as:

11 $${e}_{t}=a({h}_{t})$$

12 $${\alpha }_{t}=\frac{\text{ exp }({e}_{t})}{{\sum }_{k=1}^{T}\text{ exp }({e}_{k})}$$

13 $$ c = \sum\limits_{{t = 1}}^{T} {\alpha _{t} h_{t} }  $$

where $a$ refers to the learning function, and it can only define as ${h}_{t}$. The attention process is assumed as generating a set length of embedding layer $\mathcal{C}$ of input order with computing an adaptive weighted average of order of the states $h.$ It can be attain the last representation utilized to classifier from:

14 $${h}^{*}=\text{ tanh }\left(c\right)$$

### Hyperparameter tuning using PRO algorithm

In order to adjust the learning rate, epoch count, and batch size of the ABi-GRNN technique, the PRO algorithm is employed. If learning rate is set manually too high then there may be a failure of convergence. On the other hand, if it is set too low, then convergence to a minimum could be very slow. It is projected dependent upon people wealth performances under the society^22^. Generally, the people is clustered as to two financial classes in a society. A primary group has of wealthier people (wealth has superior to average). The next group has of worse people (wealth was lesser than average). The rich economic class people attempt for extending its class gap with observed from individuals in worse economic class. During the optimized problems, all individual solution from the Poor population moves nearby the global optimum solutions from the search space by learned in the rich solution from the Rich population.

### Encode solutions

During this case, all individual’s Person or Solution from the populations are demonstrated as binary vector. The length of all individual’s solution (binary vector) was equivalent to the amount of various features. The person $\chi$ was signified as $\chi =[{\eta }_{1}, {\eta }_{2}, {\eta }_{3}, \dots , {\eta }_{n}]$ where $n$ refers to the amount of several features from a text corpus. All the place of the person or solution $\alpha$ is binary values. ${\eta }_{j}\in \{\text{0,1}\}$.

### Initial population

The subset of solutions from the existing generation is named population. The candidate solution from the population contains the rich as well as poor economic person or solution. Assume that ‘N’ refers to the size of populations. Arbitrarily, it can be produced ‘N’ solutions with arbitrary real values amongst zero and one. Next, the digitization procedure was executed to all places of individual’s solution to convert real to binary values dependent upon in Eq. (15):

15 $$ \chi _{{i,j}}  = \left\{ {\begin{array}{*{20}l}    {1,} \hfill & {\chi _{{i,j}}  > rand} \hfill  \\    {0,} \hfill & {otherwise} \hfill  \\   \end{array} } \right. $$

At this point, $rand$ refers to the arbitrary number between zero and one. The candidate solution from the populations are decided dependent upon the main function. The top portion of the populations were represented as rich economical set of people and the bottom portion of populations were represented as worse economical set of peoples. Equation (16) illustrates the vital population from the PRO technique.

16 $$PO{P}_{Main}=PO{P}_{rich}+PO{P}_{poor}$$

### Fitness function

The FF roles are an important play from the optimized issues. It computes a positive integer for representing an optimum candidate solutions. The classifier error rate was considered as minimized FF that is expressed in Eq. (17). The rich solution is minimal fitness score (error rate) and worse solution is maximum fitness score (error rate).

17 $$fitness \left({\chi }_{i}\right)=ClassifierErrorRate({\chi }_{i})=\frac{number\;of\;misclassified\;samples}{Toral\;number\;of\;samples}*100$$

### Generate new solutions

The rich people is affecting near for increasing its economical class gap with observed in individuals from the poor economic class. The worse economic class people is affecting nearby decrease its economical class gap with learned from individuals during the rich economic class to enhance its financial status. The common performance of rich as well as poor peoples are utilized for creating a novel solution. The effort of the rich solution was determined as follows.

18 $$ \chi ^{{new}}  = \chi _{{rich,i,j}}^{{old}}  + \alpha *\left[ {\chi _{{rich,i,j}}^{{{\text{old}}}}  - \chi _{{poor,best,j}}^{{old}} } \right] $$

where, $\chi$ is the length of all individual’s solution (binary vector). The movement of the poor solution was determined in Eq. (19).

19 $${\chi }_{poor,i,j}^{new}={\chi }_{poor,i,j}^{old}+\alpha *\left[\left(\frac{{\chi }_{rich,best,j}^{0}+{\chi }_{rich,mean,j}^{0}+{\chi }_{rich,worst,j}^{0}}{3}\right)-{\chi }_{poor,i,j}^{0}\right]$$

## Results

The experimental results of the PRO-DLBIDCPS technique have been tested on NSL-KDD 2015^23^ and CICIDS 2017^24^. The NSL-KDD 2015 dataset has 125,973 samples with 41 features. Also, the CICIDS 2017 dataset incorporates 2,830,743 samples with 80 features. Table 1 provides the best cost (minimal fitness score given in Eq. (18)) and number of features chosen by the AHSA-FS technique.

**Table 1:** FS Result Analysis of AHSA Technique.

Model	$Bes{t}_{cost}$	No. of elected features	Selected features
**NSLKDD 2015 dataset**			
AHSA-FS	0.05433	15	1, 4, 5, 7, 8, 10, 12, 13, 17, 20, 24, 25, 26, 30, 35
**CICIDS-2017 dataset**			
AHSA-FS	0.04311	19	1, 3, 4, 5, 6, 7, 10, 12, 14, 16, 19, 22, 26, 26, 50, 52, 60, 63, 67

**Figure 4:**
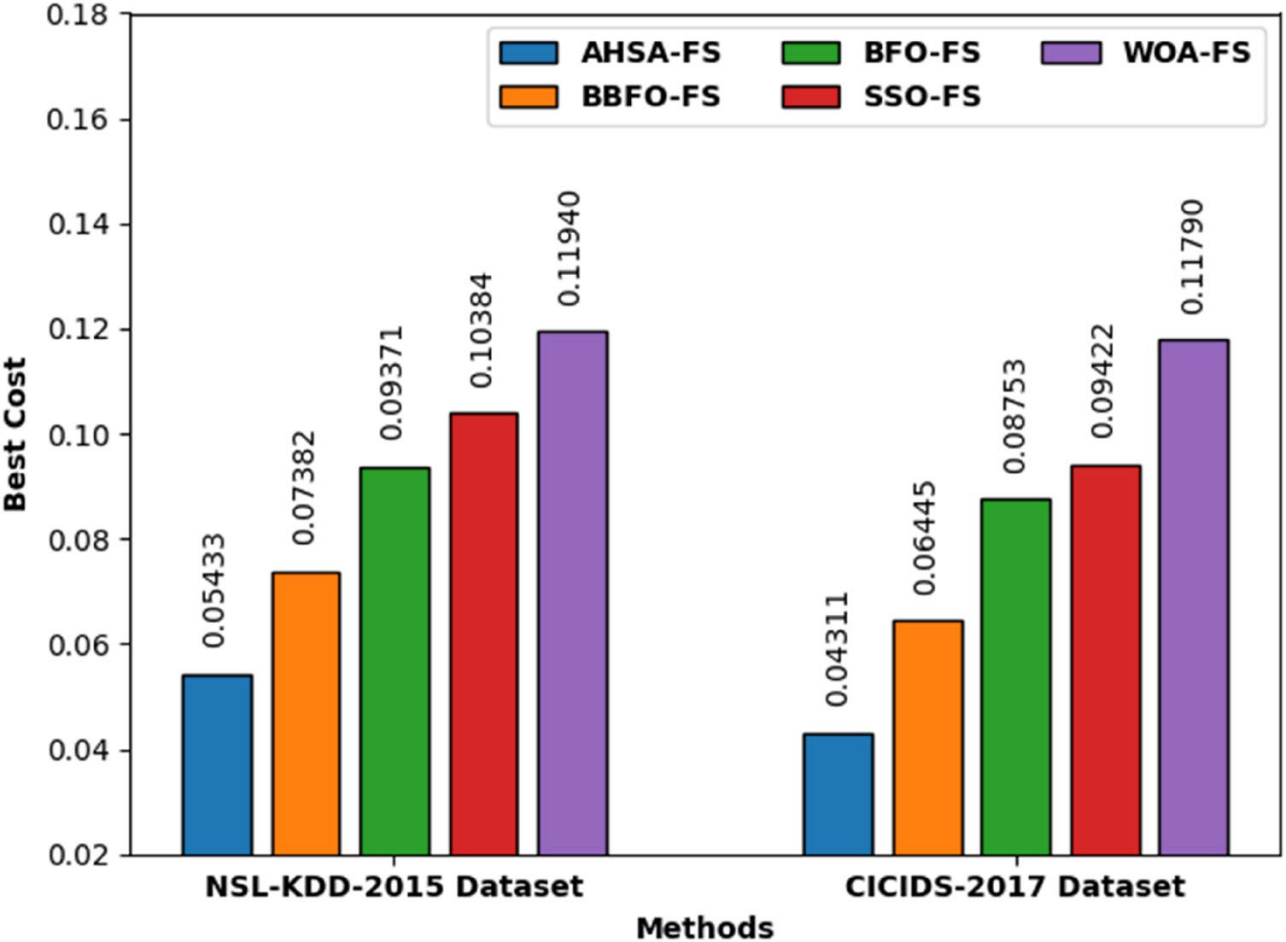
Comparative $bes{t}_{cost}$ analysis of the AHSA-FS technique.

Figure 4 showcases the best cost analysis of the AHSA-FS technique with other FS models. The results indicated that the AHSA-FS model has attained effectual outcome with least best cost under both datasets. For instance, with NLS-KDD-2015 dataset, the adaptive harmony search algorithm (AHSA-FS) model has attained minimal $bes{t}_{cost}$ of 0.05433 whereas the Binary bacterial foraging optimization (BBFOFS), Bacterial Foraging Optimization—Feature selection (BFOFS), social spider optimization—Feature Selection (SSOFS) and Whale Optimization Algorithm—Feature Selection (WOA-FS) models have reached increased $bes{t}_{cost}$ of 0.07382, 0.09371, 0.10384, and 0.1194, respectively. In addition, with CICIDS-2017 dataset, the AHSA-FS model has reached lower $bes{t}_{cost}$ of 0.04311 whereas the BBFOFS, BFOFS, SSOFS, and WOAFS models have reached maximal $bes{t}_{cost}$ of 0.06445, 0.08753, 0.09422, and 0.11790 correspondingly.

**Figure 5:**
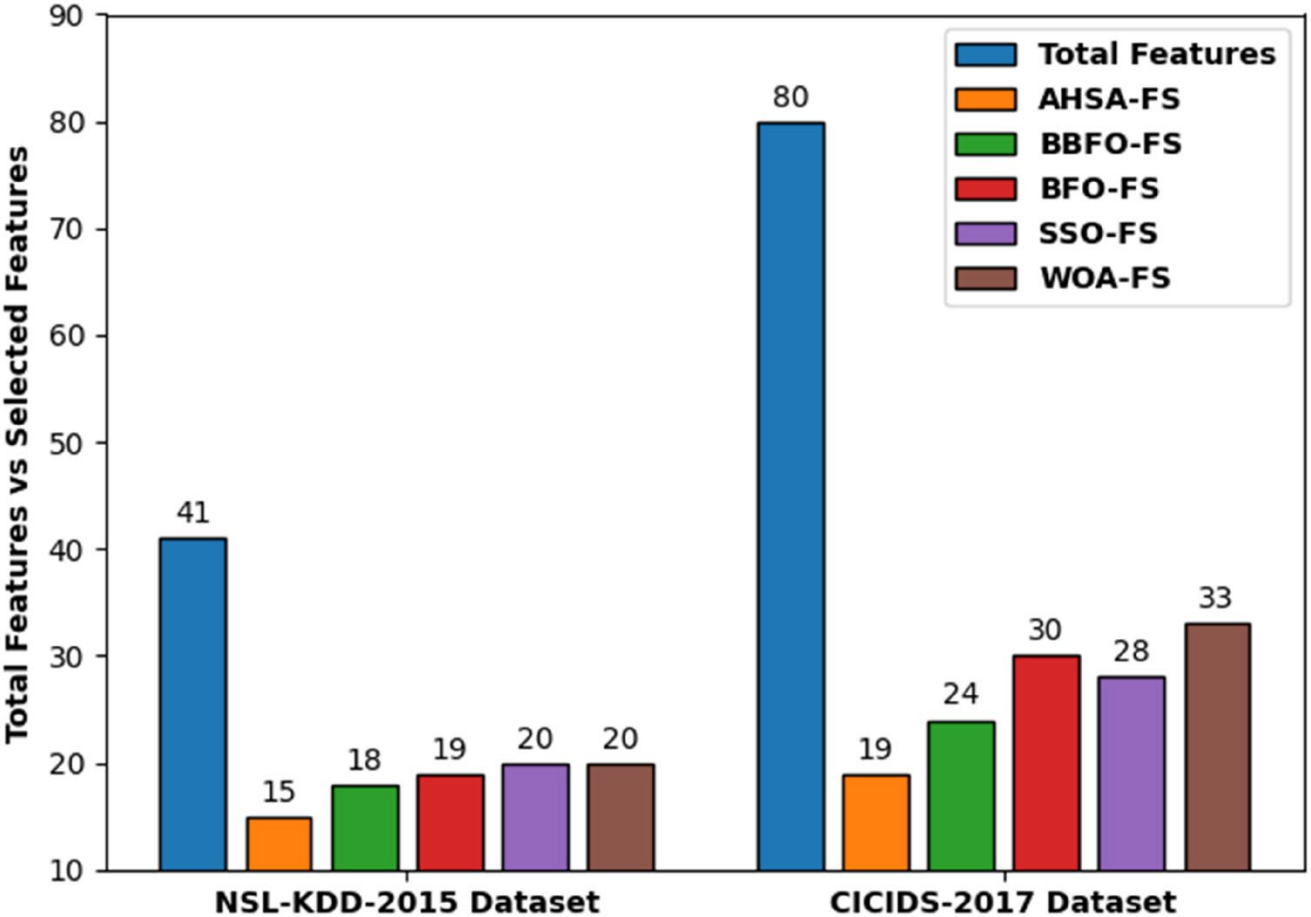
Comparative total features vs selected features of the AHSA-FS technique.

The number of features chosen by the AHSA-FS technique and other techniques is offered in Fig. 5. The results indicated that the AHSA-FS technique has chosen only minimum number of features under both datasets. For instance, with NLS-KDD-2015 dataset, the AHSA-FS technique has chosen only 15 features whereas the BBFOFS, BFOFS, SSOFS, and WOAFS techniques have elected 18, 19, 20, and 20 features respectively. Furthermore, with CICIDS 2017 dataset, the AHSA-FS approach has chosen only 19 features whereas the BBFOFS, BFOFS, SSOFS, and WOAFS methods have elected 24, 30, 28, and 33 features correspondingly.

**Figure 6:**
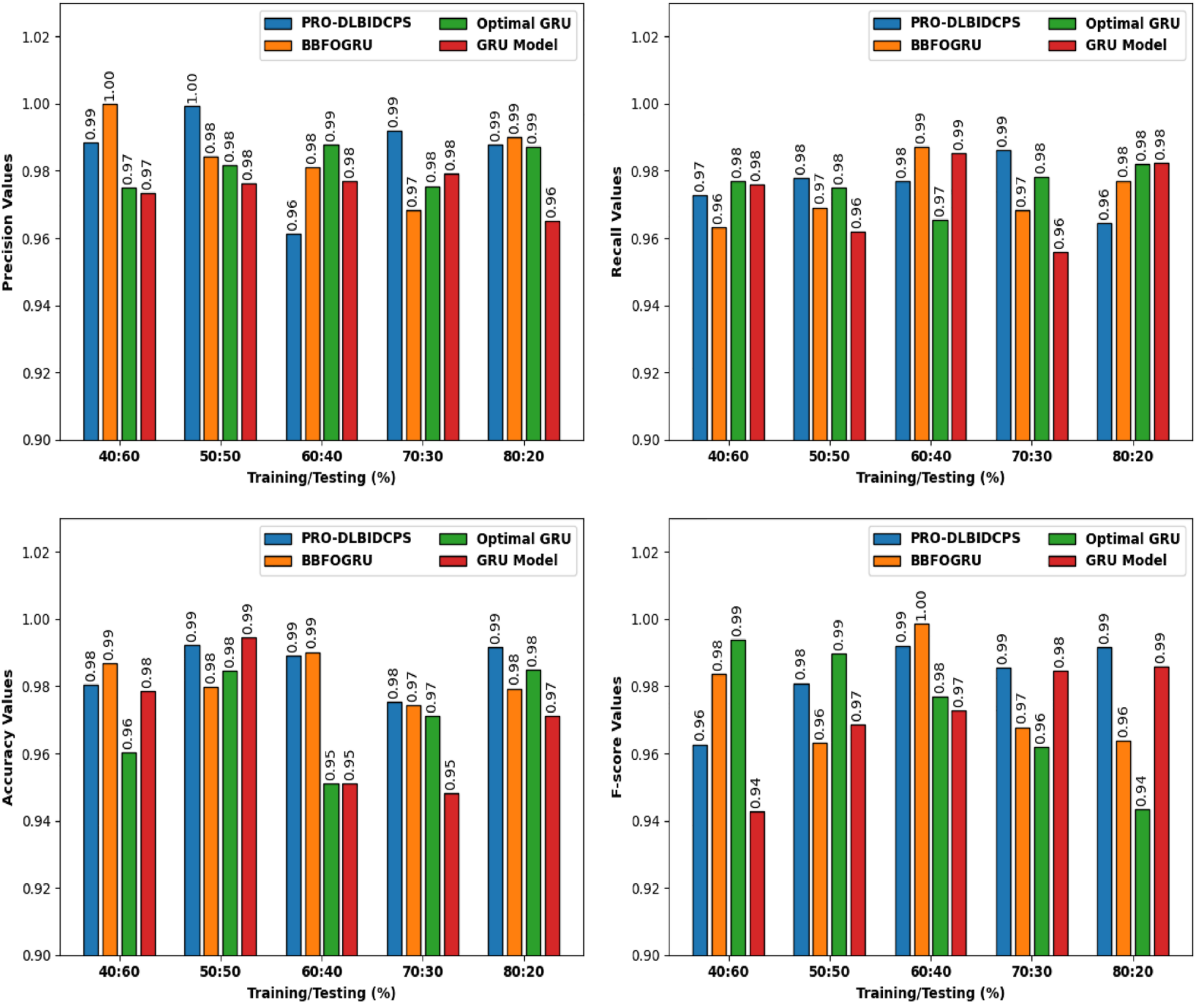
Comparative study of PRO-DLBIDCPS technique NSL-KDD-2015 dataset.

Figure 6 portrays a comprehensive comparative study of the PRO-DLBIDCPS with existing techniques on NSL-KDD-2015 dataset. The intrusion detection results are inspected under varying sets of training/testing data. The results indicated that the PRO-DLBIDCPS technique has resulted in enhanced classifier results in all aspects. For instance, with respect to precision, the PRO-DLBIDCPS technique has resulted in higher average precision (APE) of 0.9857 whereas the BBFO, gated recurrent unit (GRU), optimal GRU, and GRU approaches have reached reduced APE values of 0.9847, 0.9814, and 0.9741 respectively. Meanwhile, with respect to recall, the PRO-DLBIDCPS technique has resulted in higher average recall (ARE) of 0.9757 while the BBFO-GRU, optimal GRU, and GRU methods have obtained lesser WOAFS values of 0.9730, 0.9755, and 0.9723 correspondingly. Eventually, in terms of accuracy, the PRO-DLBIDCPS system has resulted in higher average accuracy (AAC) of 0.9858 while the BBFOGRU, optimal GRU, and GRU techniques have gained minimum AAC values of 0.9821, 0.9704, and 0.9687 respectively. Lastly, with respect to F-score, the PRO-DLBIDCPS technique has resulted in superior average F-score of 0.9826 but the BBFOGRU, optimal GRU, and GRU methods have obtained reduced average F-score values of 0.9755, 0.9732, and 0.9709 correspondingly. 100% precision values have been achieved by BBFOGRU and PRO-DLBIDCPS as training dataset is less or equal than testing dataset. It is due to ratio of training and testing datasets.

**Figure 7:**
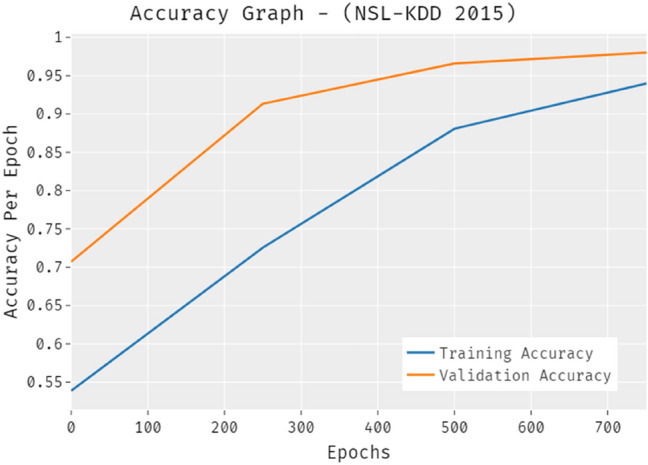
Accuracy analysis of PRO-DLBIDCPS technique NSL-KDD-2015 dataset.

The accuracy outcome inspection of the PRO-DLBIDCPS technique on NSL-KDD-2015dataset is portrayed in Fig. 7. The results demonstrated that the PRO-DLBIDCPS technique has accomplished improved validation accuracy compared to training accuracy. It is also observable that the accuracy values get saturated with the count of epoch.

**Figure 8:**
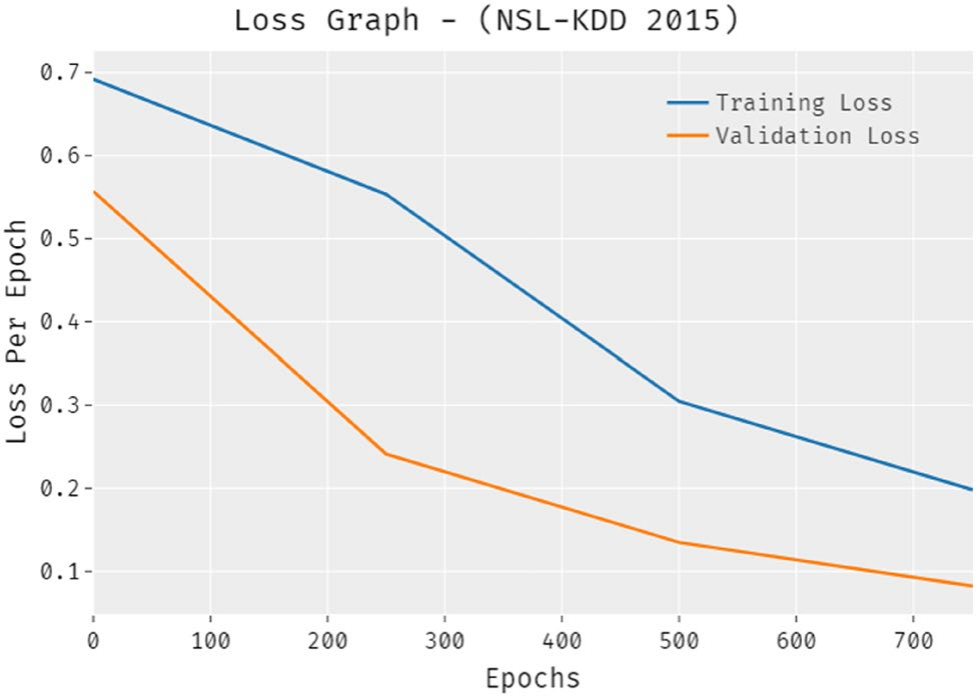
Loss analysis of PRO-DLBIDCPS technique NSL-KDD-2015 dataset.

The loss outcome investigation of the PRO-DLBIDCPS technique on NSL-KDD-2015dataset is depicted in Fig. 8. The results revealed that the PRO-DLBIDCPS approach has denoted the reduced validation loss over the training loss. It is additionally noticed that the loss values get saturated with the epoch count of epoch.

**Figure 9:**
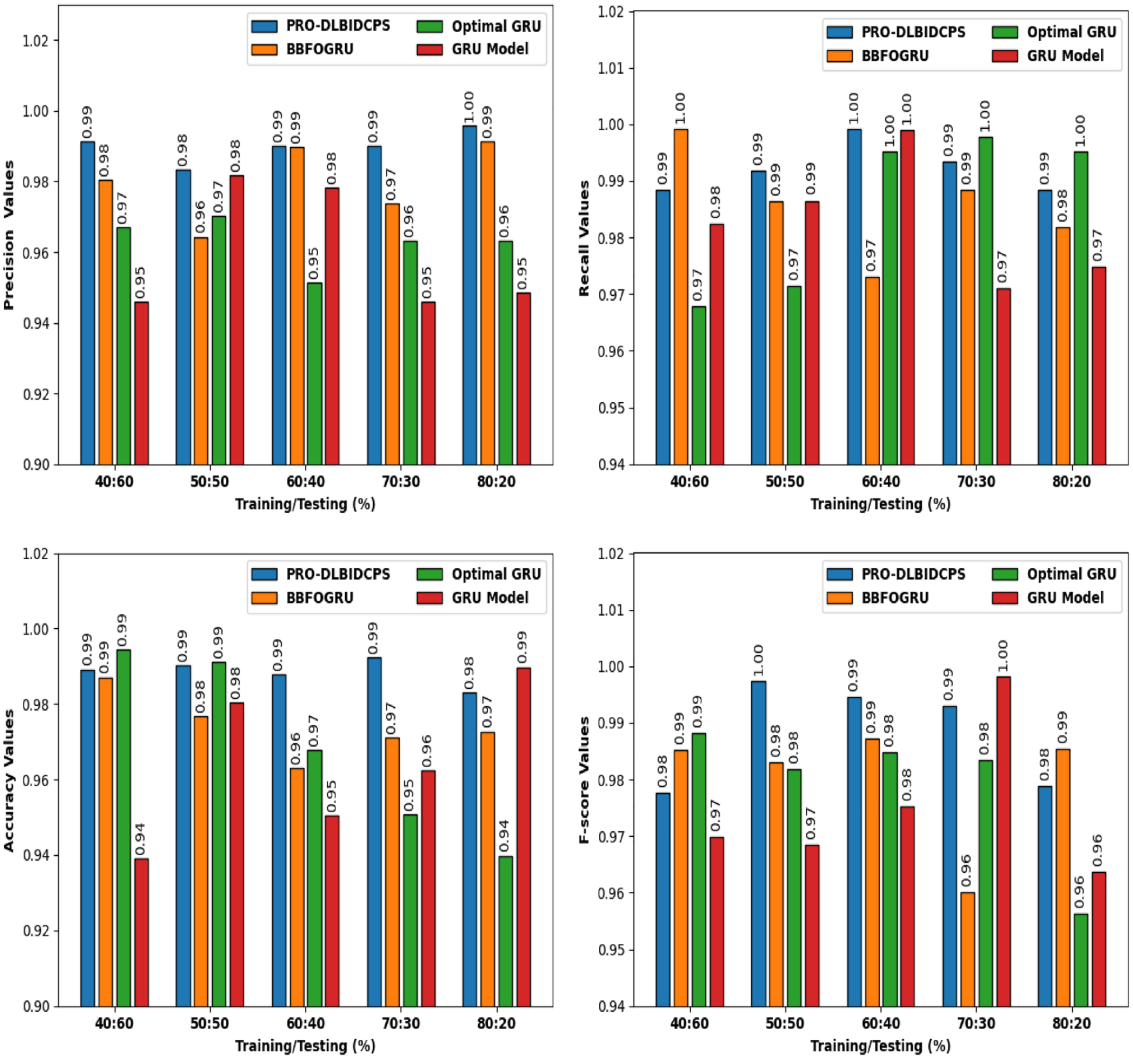
Comparative study of PRO-DLBIDCPS technique CICIDS-2017 dataset.

Figure 9 depicts a comprehensive comparative study of the PRO-DLBIDCPS with existing approaches on CICIDS-2017 dataset. The intrusion detection results are inspected under varying sets of training/testing data. The outcomes show that the PRO-DLBIDCPS system has resulted in enhanced classifier results in all aspects. For sample, with respect to precision, the PRO-DLBIDCPS approach has resulted in higher APE of 0.9901 whereas the BBFOGRU, optimal GRU, and GRU models have provided least APE values of 0.9799, 0.9630, and 0.9600 respectively. In the meantime, with respect to recall, the PRO-DLBIDCPS technique has resulted in higher WOAFS of 0.9922 while the BBFOGRU, optimal GRU, and GRU methodologies have gained decreased WOAFS values of 0.9858, 0.9854, and 0.9828 correspondingly.

Followed by, with respect to accuracy, the PRO-DLBIDCPS algorithm has resulted in higher AAC of 0.9885 whereas the BBFOGRU, optimal GRU, and GRU methods have obtained lower AAC values of 0.9741, 0.9687, and 0.9644 respectively. At last, with respect to F-score, the PRO-DLBIDCPS system has resulted in superior average F-score of 0.9883 whereas the BBFOGRU, optimal GRU, and GRU models have obtained lower average F-score values of 0.9802, 0.9789, and 0.9751 correspondingly.

**Figure 10:**
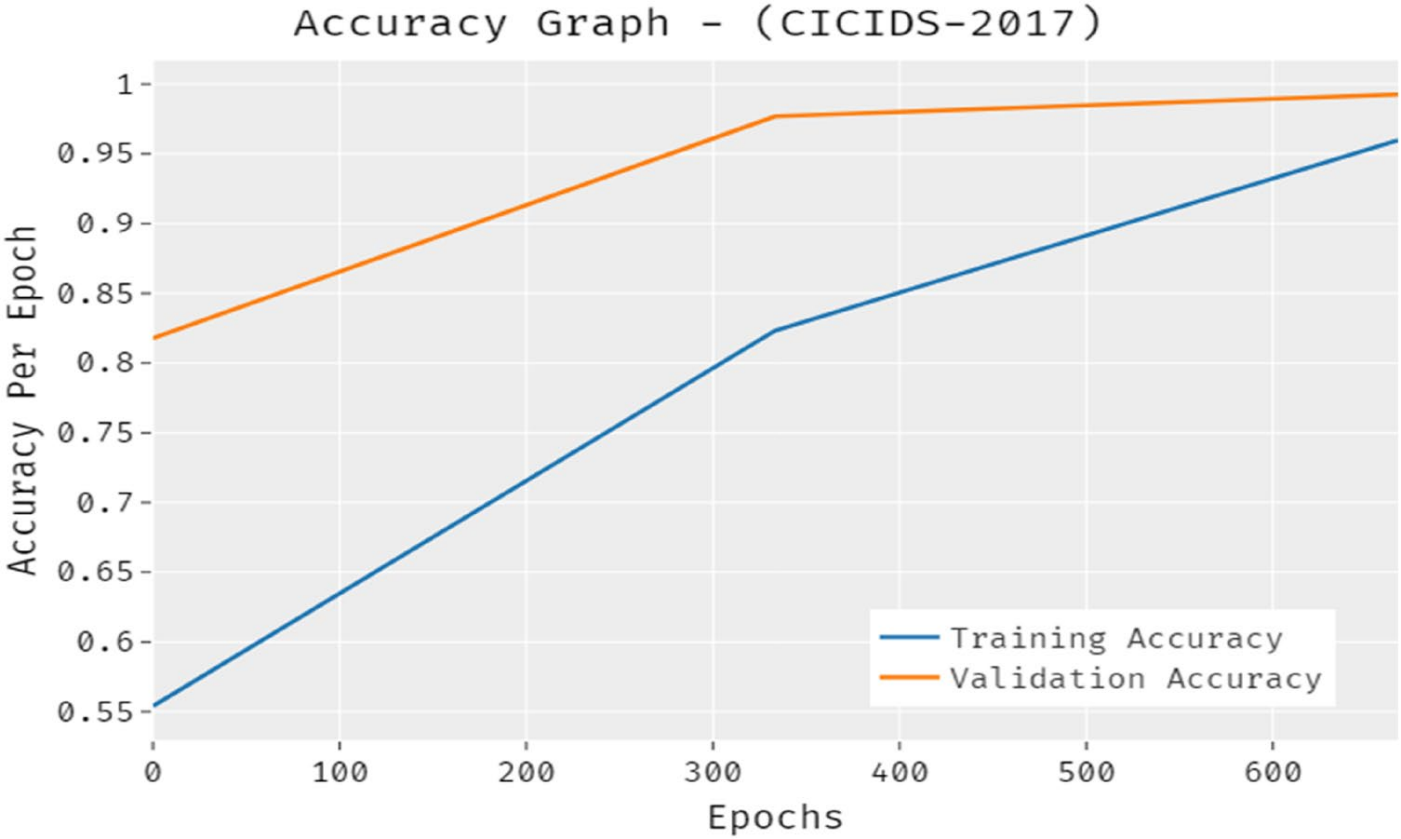
Accuracy analysis of PRO-DLBIDCPS technique CICIDS-2017 dataset.

The accuracy outcome analysis of the PRO-DLBIDCPS approach on CICIDS-2017 dataset is illustrated in Fig. 10. The results exhibited that the PRO-DLBIDCPS system has accomplished improved validation accuracy compared to training accuracy. It is also observable that the accuracy values get saturated with the count of epoch.

**Figure 11:**
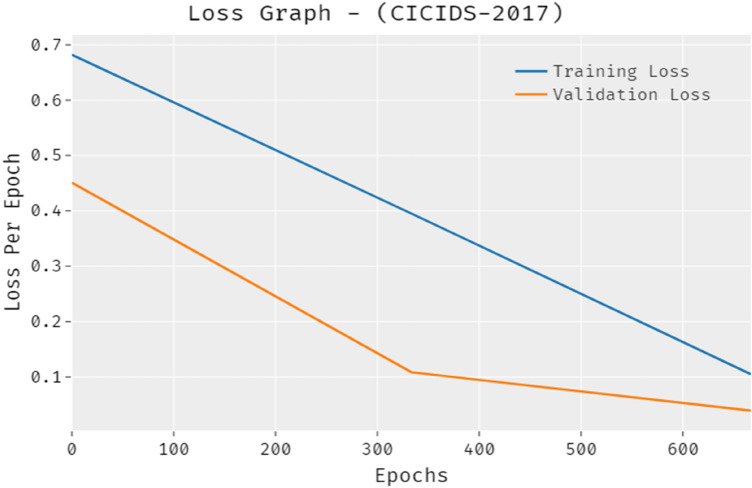
Loss analysis of PRO-DLBIDCPS technique CICIDS-2017 dataset.

The loss outcome analysis of the PRO-DLBIDCPS method on CICIDS-2017 dataset is depicted in Fig. 11. The figure exposed that the PRO-DLBIDCPS algorithm has denoted the lower validation loss over the training loss. It can be additionally noticed that the loss values were saturated with the epoch count of epoch.

**Figure 12:**
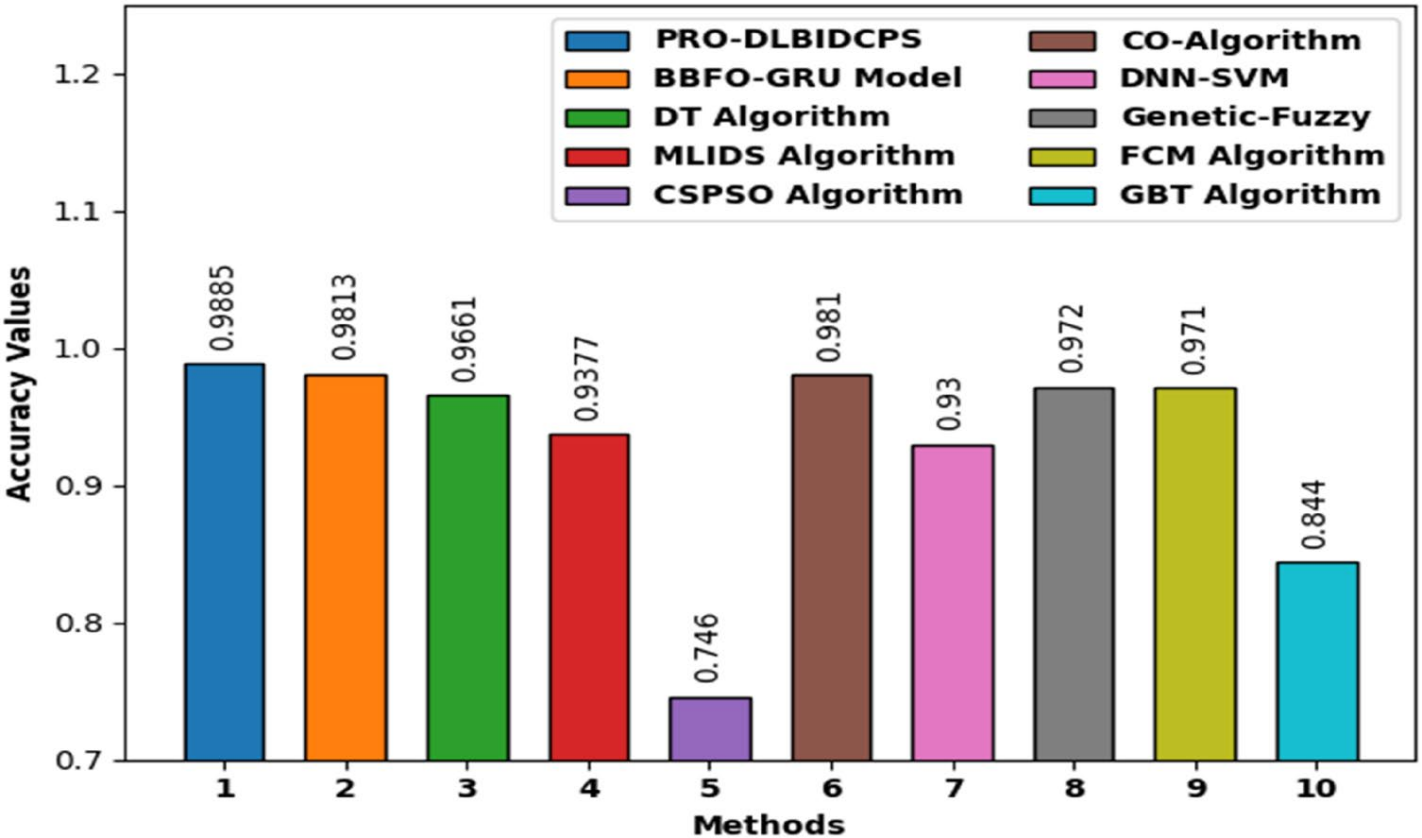
Comparative accuracy analysis of the PRO-DLBIDCPS technique.

The performance of the PRO-DLBIDCPS technique can be compared with recent methods^25^ in terms of $ACC{U}_{Y}$ in Fig. 12 and Table 2. The results indicated that the chaotic particle swarm optimization (CSPSO) algorithm has resulted in least $ACC{U}_{Y}$ of 0.7460. At the same time, the Multi layers intrusion detection system (MLIDS) and deep neural network support vector machine (DNN-SVM) models have resulted in slightly enhanced $ACC{U}_{Y}$ of 0.9377 and 0.9300 respectively. Along with that, the Decision Tree (DT), CO-algorithm, Genetic-Fuzzy, and Fuzzy C Means (FCM) models have attained moderately increased $ACC{U}_{Y}$ of 0.9661, 0.9810, 0.9720, and 0.9710 respectively. Though the BBFO-GRU technique has resulted in near optimal $ACC{U}_{Y}$ of 0.9885, the proposed PRO-DLBIDCPS technique has outperformed the other ones with the higher $ACC{U}_{Y}$ of 0.9885.

**Table 2:** Comparative study of PRO-DLBIDCPS technique in terms of accuracy.

Methods	Accuracy (%)
PRO-DLBIDCPS	0.988
BBFO-GRU Model	0.981
DT Algorithm	0.966
MLIDS Algorithm	0.937
CSPSO Algorithm	0.746
CO-Algorithm	0.981
DNN-SVM	0.930
Genetic-Fuzzy	0.972
FCM Algorithm	0.971
GBT Algorithm	0.844

**Figure 13:**
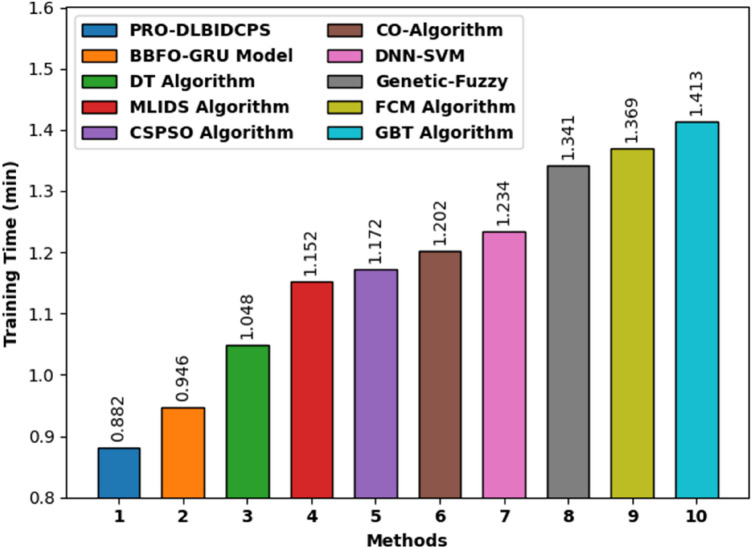
Comparative TGT analysis of the PRO-DLBIDCPS technique.

Table 3 provides the time complexity analysis of the PRO-DLBIDCPS technique with recent approaches in terms of training time (TGT) and testing time (TST). Figure 13 reports the TGT examination of the PRO-DLBIDCPS with existing techniques. The results reported that the Gradient Boosting based Tree (GBT), FCM, Genetic-Fuzzy, DNN-SVM, CO algorithm, CSPSO, and MLIDS techniques have attained higher TGT of 1.413 min, 1.369 min, 1.341 min, 1.234 min, 1.202 min, 1.172 min, and 1.152 min, respectively. Followed by, the DT and BBFO-GRU techniques have reached moderately TGT of 1.048 min and 0.946 min, respectively. However, the PRO-DLBIDCPS technique has resulted in effectual outcome with the least TGT of 0.882 min.

**Table 3:** Comparative study of PRO-DLBIDCPS technique in terms of TST and TGT.

Methods	Training time (min)	Testing time (min)
PRO-DLBIDCPS	0.882	0.330
BBFO-GRU Model	0.946	0.393
DT Algorithm	1.048	0.547
MLIDS Algorithm	1.152	0.541
CSPSO Algorithm	1.172	0.565
CO-Algorithm	1.202	0.572
DNN-SVM	1.234	0.596
Genetic-Fuzzy	1.341	0.674
FCM Algorithm	1.369	0.693
GBT Algorithm	1.413	0.725

**Figure 14:**
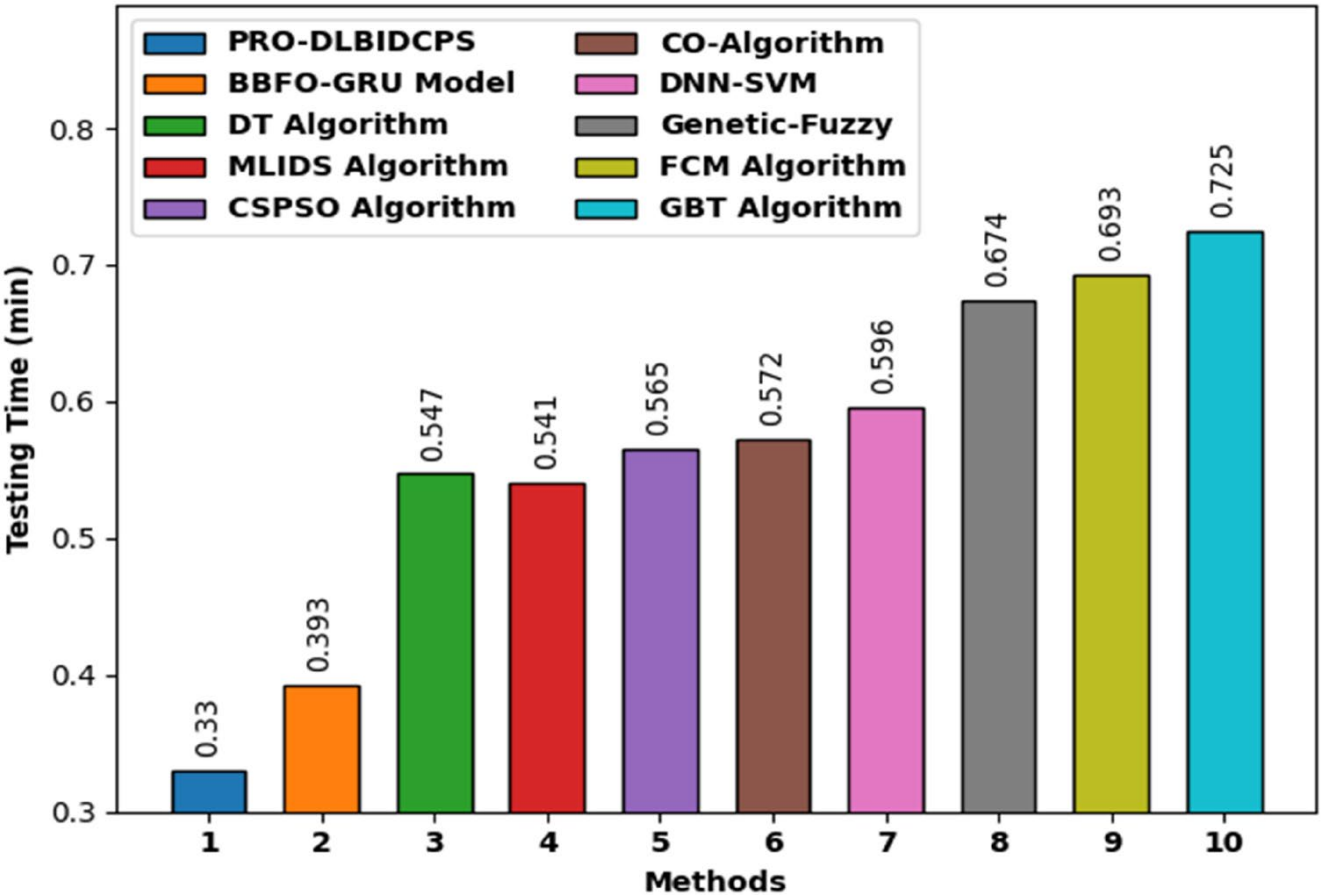
Comparative TST analysis of the PRO-DLBIDCPS technique.

Figure 14 examines the TST examination of the PRO-DLBIDCPS with existing approaches. The outcomes stated that the GBT, FCM, Genetic-Fuzzy, DNN-SVM, CO algorithm, CSPSO, DT, and MLIDS techniques have reached maximum TST of 0.725 min, 0.693 min, 0.674 min, 0.596 min, 0.572 min, 0.565 min, 0.547 min, and 0.541 min correspondingly. Then, the BBFO-GRU systems have reached to considerably lower TST of 0.393 min. Lastly, the PRO-DLBIDCPS methodology has resulted in effectual outcome with the least TST of 0.33 min.

From the above mentioned results, it is clear that the PRO-DLBIDCPS technique has accomplished maximum intrusion classification outcome in the CPS environment.

## Conclusion

In this study, a new PRO-DLBIDCPS technique has been developed for intrusion detection in the CPS environment. The PRO-DLBIDCPS technique encompasses different processes namely pre-processing, AHSA for election of features, ABi-GRNN classifier, and PRO hyperparameter optimizer. The detection efficiency of the ABi-GRNN technique has been enhanced by the use of PRO algorithm based hyperparameter optimizer, which results in enhanced intrusion detection results. Also, the inclusion of blockchain technology helps in enhancing security in the CPS environment. A wide ranging simulation analysis is performed to ensure the enhanced performance of the PRO-DLBIDCPS technique in terms of several measures. The comprehensive comparative results reported the better outcomes of the PRO-DLBIDCPS technique in terms of several measures. In future, data clustering and feature reduction techniques can be integrated to the PRO-DLBIDCPS technique for accomplishing maximum security in the CPS environment.

## Data Availability

Data is available on http://www.unb.ca/research/iscx/dataset/iscx-NSL-KDD-dataset.html and https://www.unb.ca/cic/datasets/ids-2017.html for experimental purposes.
